# Cryptosporidiosis in a Zoonotic Gastrointestinal Disorder Perspective: Present Status, Risk Factors, Pathophysiology, and Treatment, Particularly in Immunocompromised Patients

**DOI:** 10.1155/2024/6439375

**Published:** 2024-11-05

**Authors:** Thivya Balendran, Devika Iddawela, Sajanee Lenadora

**Affiliations:** Department of Parasitology, Faculty of Medicine, University of Peradeniya, Peradeniya 20400, Sri Lanka

## Abstract

*Cryptosporidium* infection is highly prevalent among immunocompromised patients with Acquired Immunodeficiency Syndrome, cancer, primary immunodeficiency, and organ transplant recipients. Comprehensive knowledge about *Cryptosporidium* infection provides the means for efficient diagnosis, treatment, and prevention. Therefore, with the objective of providing an in-depth analysis of Cryptosporidiosis in immunocompromised patients, this review presents a comprehensive understating of the prevalence, risk factors, pathophysiology of *Cryptosporidium* infection, clinical presentation in the immunocompromised, the immune response of the host, diagnostic methods performed in laboratory settings, possible treatments, and prevention methods, which can be used for further studies. Peer-reviewed, published, original articles on cryptosporidiosis in immunocompromised patients were searched using specific key-words on PubMed, ResearchGate, Google Scholar, and ScienceDirect databases. Articles which were accessible to the date of 18^th^ of August 2023, were included in this comprehensive review. We analyzed reports on *Cryptosporidium* in immunocompromised patients with human immunodeficiency virus/acquired immunodeficiency syndrome (HIV/AIDS), cancer, primary immunodeficiency, and organ transplant recipients. 134 Articles describing epidemiology, related risk factors, clinical presentation, diagnosis, and possible treatments in the light of pathogenesis, pathophysiology, and virulence factors of *Cryptosporidium* and immunology of the host are summarized in this study. Effective treatments to be administered, importance, and ways of prevention were identified. *Cryptosporidium* infection was found to be highly prevalent among immunocompromised in Asia, Africa, Europe, and North America. The immunity of the host and the decrease in CD4^+^ T-cell count were found to the main factors which decide the susceptibility and the severity of infection. Drugs that activate host immunity and suppress *Cryptosporidium* growth, along with supportive therapy, is an effective treatment. But prevention is the most effective strategy for immunocompromised patients; thus, a better understanding about the disease would lead to effective prevention.

## 1. Introduction

Cryptosporidiosis is caused by *Cryptosporidium* species, an obligate intracellular parasite and an emerging opportunistic pathogen in immunocompromised patients [1, 2]. Infection in humans is caused by *Cryptosporidium hominis* and *Cryptosporidium parvum*. Transmission is by oocysts via contaminated food and water and person to person spread [3]. It causes diarrhea worldwide and severe disease in immunocompromised individuals especially in patients with human immunodeficiency virus/acquired immunodeficiency syndrome (HIV/AIDS), cancer, primary immunodeficiency, and individuals who have undergone organ transplants [1, 4–6]. It has been estimated that 7.5 million new cases of cryptosporidium infection occur yearly. According to the Global Burden of Disease study, acute and long-term effects of cryptosporidiosis amount to a loss of 8.2 million DALYs (Disability-Adjusted Life Years) [7]. A high prevalence of *Cryptosporidium* infection in HIV patients is recorded in Asia and Africa and organ transplant recipients in Asia, Europe, Africa, and North America. Cancer became highly prevalent within the last decade and treatment such as chemotherapy or bone marrow transplantation for cancer makes patients susceptible of *Cryptosporidium* infection [8]. The global burden of *Cryptosporidium* in immunocompromised patients is increasing, and possible measures should be taken in terms of identifying risk factors, diagnosis, treatments, and prevention. Therefore, with the objective of providing an in-depth analysis of cryptosporidiosis in immunocompromised, this review presents a comprehensive understating of the risk factors, pathophysiology of *Cryptosporidium*, the immune response of the host, clinical presentation observed among immunocompromised individuals, diagnostic methods performed in laboratory settings, possible treatments, and prevention methods, which can be used for further studies.

## 2. Materials and Methods

**Figure 1:**
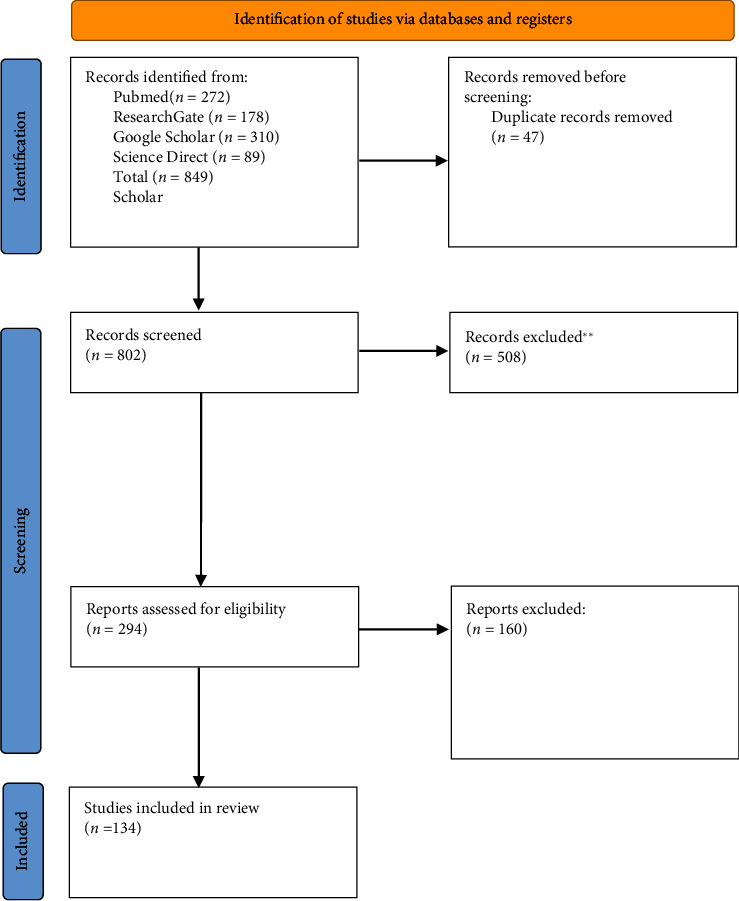
PRISMA 2020 flow diagram for new systematic reviews [9].

Peer-reviewed, published, original articles on Cryptosporidiosis in immunocompromised patients were searched on PubMed, ResearchGate, Google Scholar, and ScienceDirect databases. Following keywords were used for the search: “*Cryptosporidium* infection,” “cryptosporidiosis in immunocompromised patients,” “prevalence of cryptosporidiosis in immunocompromised patients,” “risk factors of cryptosporidiosis,” “Diagnosis, treatment, and prevention of cryptosporidiosis,” and “pathogenicity and virulence of cryptosporidiosis.” Articles which were accessible to the date of 18^th^ of August 2023, were included in this comprehensive review. Full-text articles available in English were included while restricted articles and articles which were unavailable in English were excluded. The reviewers screened the articles independently and tabulated results of individual studies [9] Figure 1.

## 3. Results and Discussion

### 3.1. Prevalence

*Cryptosporidium* infection is distributed around the globe. It is estimated that 7.6 million cases of *Cryptosporidium* spp. are identified annually while 48,000 to 202,000 deaths occur, mainly among young children in under-privileged settings [10, 11]. Cryptosporidiosis prevalence is significantly higher in developing countries compared to developed countries [12]. Humans can be infected by zoonotic and nonzoonotic means such as ingesting parasite oocysts from contaminated water and food [13]. In addition to fecal-oral transmission, Cryptosporidiosis can be transmitted through inhalation or coughing and infect the respiratory tract in immunosuppressed patients [10]. *Cryptosporidium* infection in HIV infected patients was reported mainly in Asia and Africa, with the prevalence of 13.1%–60.4% in India, 30.3% in Thailand, 22.5% in Ethiopia, 19.2% in Nigeria, 6%–10.8% in Iran, and 5.4% in Congo [14–22]. A higher prevalence of 30% was recorded in France [23] (Supporting table [5, 24–36]). *Cryptosporidium parvum*, *Cryptosporidium hominis,* and *Cryptosporidium meleagridis* have been found in immunocompromised patients (Supporting table [5, 24–36]).

*Cryptosporidium* infection in organ transplant recipients is reported in Asia, Europe, Africa, and North America with a prevalence of 38.0% in the USA, 0.3%–24% in Iran, 22.5% in Turkey, 18.3% in Hungary, 8.4% in India, and 0.8% in Sudan [23, 37–43]. Frequently transplanted organ was found to be the kidney in most of the studies along with liver, heart, and pancreases. Cryptosporidiosis in cancer patients shows a prevalence of 32.0% in Egypt, 21.0% in Lebanon, 12.6%–18% in Poland, 13.3% in China, and 2.2%–4.2% in Iran [5, 13, 20, 41, 44–47]. The infection is frequently reported with colorectal cancer in most of the studies [5]. Cryptosporidiosis is also reported in primary immunodeficient patients in Iran and the Netherlands with a prevalence of 7.5% and 66.7%, respectively [4, 37]. These patients had X-linked hyper-IgM Syndrome (XHIM) and primary CD4 lymphopenia. Studies carried out in Sri Lanka report *Cryptosporidium* infection in children and animals, but not in immunocompromised patients.

### 3.2. Risk Factors

Various studies reported *Cryptosporidium* infection and related risk factors associated with infection in immunocompromised patients. Studies report seasonal variation (high rainfall), CD4^+^ T-cell count < 200 cells/*μ*L, animal contact (calf), diarrhea in family members, and antiviral therapy as risk factors found among HIV/AIDS patients [15, 18–20, 23, 48]. The immunity of the host is the main host factor that decides the probability and the severity of infection [49]. Immune suppression that affects T-cell function leads to increased severity of disease [50]. But deficiencies that affect B cell function are not associated with *Cryptosporidium* infection [51]. For HIV patients CD4^+^ cell counts < 50, leads to a severe disease state [52]. For organ transplant recipients following immunosuppressing drugs, seasonal variation, traveling to Cryptosporidiosis endemic areas, living in rural areas, animal contact (farm animals), consuming non-potable water, using recreational water, and HIV infection are reported as risk factors [23, 39, 41]. Studies determined that hygiene status, living area, diarrhea, education level, age below 20 years, male gender, CD4^+^ T-cell count < 200 cells/*μ*L, bone marrow transplantation, and family members with diarrhea as associated risk factors among cancer patients [5, 20, 44, 53]. A study carried out in Sri Lanka reported a 5.7% *Cryptosporidium* infection prevalence in children aged below 3 years with possible risk factors as animal contact and usage of unboiled drinking water [54].

### 3.3. Pathogenesis

**Figure 2:**
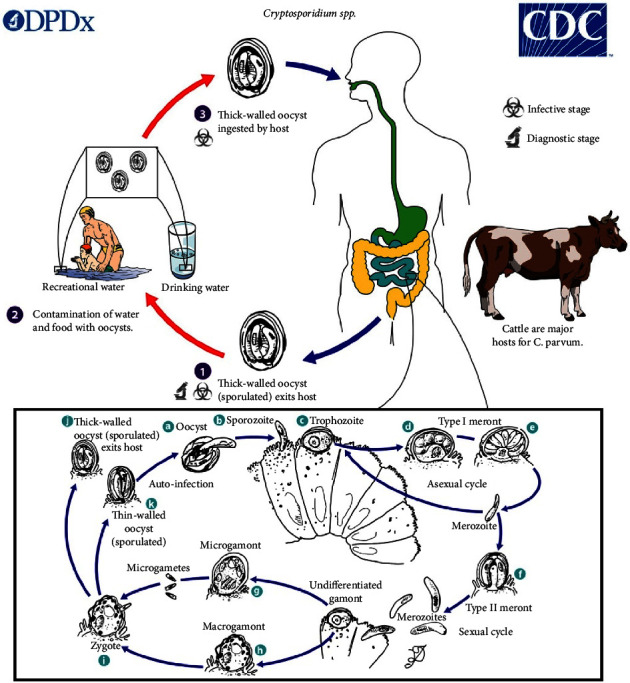
*Cryptosporidium* spp. life cycle [61].

*Cryptosporidium*, an intracellular parasite, established on the apical surface membrane-bound compartment of intestinal epithelium intracellularly, but in an extra-cytoplasmic vacuole [55, 56]. Normally it does not cause deep penetration or systematic infection. Upon ingestion of the infected oocysts, sporozoites are released in the intestines (terminal ileum and jejunum). The initial attachment of sporozoites to the epithelial cell is followed by the formation of the parasitophorous vacuole in intracellular extra-cytoplasmic space [3]. An electron-dense feeder stage called trophozoite is formed for energy and nutrient acquisition. Asexual replication of sporozoites occurs inside the developing schizont and is released as merozoites from a mature schizont, which infect surrounding epithelial cells in the intestine. The sexual replication occurs by macrogamete and microgamete fusion and formation of zygotes, which form thick or thin wall oocysts with four sporozoites by two asexual cycles [57]. Thick-walled oocysts are capable of withstanding environmental conditions and are transported and excreted in feces [58, 59]. Thin-walled oocysts separated from the epithelium enable autoinfection, leading to chronic infection [57, 60, 61] Figure 2.

### 3.4. Pathophysiology

This infection commonly produces watery diarrhea and malabsorption. It disrupts the absorption, permeability, and secretion of electrolytes and fluid. It damages the host epithelial cells directly or through cytokines and inflammatory cells at the site of infection [55]. Depending on the host immune state, the severity and persistence of the outcome varies [62]. A secretory component due to increased P substance or production of prostaglandin can be seen and disruption of the intestinal epithelial cells can inhibit the absorption of NaCl [63, 64]. In addition, the infection may contribute to apoptosis of adjacent epithelial cells and establish an extended parasite survival by inhibiting apoptosis of the infected cells [65]. Even though primary infection is caused in the small intestine, *Cryptosporidium* can infect the biliary tract, pancreas, and lungs of immunocompromised patients [1].

### 3.5. Virulence Factors of *Cryptosporidium*

Virulence factors are thought to be substances and processes that enable the parasite to initiate and continue the disease state in hosts. Unless the parasite inside the body is completely killed, virulence factors will affect the host at any point [66]. Different degrees of virulence within *Cryptosporidium* species and various levels of host susceptibility toward infection have been reported in many studies. More than 25 virulence factors have been identified for *Cryptosporidium* infection, which involve the interaction between host and pathogen, in terms of adhesion to proliferation [49].

### 3.6. Clinical Presentation

#### 3.6.1. Immunocompetent Patients

Clinical presentation of *Cryptosporidium* infection depends on the immunity level of individuals. Cryptosporidium is a common cause of self-limiting diarrhea among immunocompetent people [37]. Children aged less than 1 year and elderly population, mostly in developing countries are infected by *Cryptosporidium*, where the infection can be asymptomatic, persistent, or acute [1, 2]. Clinical presentation of immunocompetent patients was identified in previous studies as increased stool frequency in a day, nonbloody diarrhea, vomiting, anorexia, abdominal pain, mild fever, dehydration, fatigue, weight loss, and a short period of watery diarrhea up to 2 weeks [37, 67]. A study done by Lee et al. shows that peripheral blood C-reactive protein levels, neutrophil proportions, and Erythrocyte Sedimentation Rates were high in immunocompetent individuals [68].

#### 3.6.2. Immunocompromised Patients

##### 3.6.2.1. HIV Patients

Patients with HIV/AIDS show gastrointestinal disease as the most common clinical presentation, and diarrhea has been identified as an AIDS defining condition [14]. They develop profuse diarrhea like cholera with malabsorption and fluid depletion. Chronic diarrhea, transient diarrhea, relapsing illness, and Cholera-like disease are reported as clinical syndromes in *Cryptosporidium* positive AIDS patients in a study carried out in the USA. The same study reports a higher rate of survival in *Cryptosporidium* negative AIDS patients compared to the *Cryptosporidium* infected AIDS patients [69]. Along with these, HIV infected patients can develop a severe type of extraintestinal presentation of Cryptosporidiosis that includes atypical gastrointestinal disease, acalculous cholecystitis, biliary tract disease, pancreatitis, and respiratory tract disease as a primary intestinal infection extension [1, 50, 70, 71]. Differences can be seen in clinical manifestations among *Cryptosporidium* species in HIV patients [15]. A study reported a significant association between recurrent abdominal pain and HIV infection [16]. The risk of clinical symptoms is increased along with the decreasing CD4^+^ T-lymphocytes, where in a study conducted in Nigeria, was CD4^+^ less than 350 [72].

##### 3.6.2.2. Organ Transplant Patients

Patients who have undergone various organ transplants that include kidney, liver, pancreas, heart, liver-kidney, pancreas-kidney, and bowel, reported *Cryptosporidium* infection during the post-transplant period [23, 38, 39]. Immunosuppressive drug therapy was found to be the main reason for this [6]. A case series in France reported patients who underwent renal transplants infected with *Cryptosporidium* with the following clinical presentations: watery diarrhea, vomiting, nausea, asthenia, hypotension, weight loss, dry mouth, abdominal pain, esophageal pain, and acute renal failure [73]. Lanternier et al. [39] reported that *Cryptosporidium* infection occurs at a median period of 3.4 (0–19.8) years post-transplant. Co-infection of multiple parasites including *C.belli, Cryptosporidium cayetanensis, Giardia lamblia, Blastocystis hominis, Hymenolepis nana,* and *Strongyloides stercoralis* were reported in other studies [6].

##### 3.6.2.3. Cancer Patients

Diarrhea, vomiting, poor appetite, loss of weight, abdominal distension, and moderate dehydration was observed in Acute Lymphoblastic Leukemia [74]. In another study, difficulty with defecation, flatulence, unintended weight loss, abdominal pain, and increased fatigue were observed, and *C. meleagridis* was identified in a colorectal cancer patient [75]. Another study revealed watery diarrhea, mucus, and soft stool, in leukemia, neuroblastoma, lymphoma, and brain cancer in paediatric patients [76]. Individuals with haematological malignancies showed clinical presentation similar to solid organ malignancies. Severe infection showed *Cryptosporidium* oocysts shedding for 49 days [77]. Furthermore, it was postulated in 2022 that *Cryptosporidium* spp. might lead to cause mucosal dysplasia in colon, thereby leading to development of colonic cancer [78].

##### 3.6.2.4. Primary Immunodeficiency Patients

A study carried out by Wolska-Kusnierz et al. [4] reports *Cryptosporidium* infection with XHIM Syndrome type 1 and primary CD4 lymphopenia. Patients with CD4 lymphopenia showed stunted growth, malnutrition, mucosal atrophy, and chronic diarrhea, along with bile duct inflammation and sclerosing cholangitis [4]. Patients with XHIM syndrome did not show malnutrition and chronic diarrhea but developed bile duct inflammation, sclerosing cholangitis, and liver disease due to *Cryptosporidium* infection. Studies report Cryptosporidiosis in Hematopoietic Stem Cell Transplant (HSCT) recipients and Dedicator of Cytokinesis 8 (DOCK 8) deficiency patients [79–81]. Another study reports recurrent diarrhea and sclerosing cholangitis caused by *Cryptosporidium* as the clinical presentation [82].

### 3.7. Immunology

Adaptive and innate immune responses against *Cryptosporidium* infection play an important role in protecting the host from Cryptosporidiosis [83]. The epithelial cells of the intestine serve as the host cell and facilitate parasite replication and promote protective immunity by activating nuclear factor-kappa B signal pathway [84]. Epithelial cells produce chemokines which belong to CXC and CC families (CCL2, CCL5, CXCL9, CXCL10) which attract the immune cells to the area of infection. They induce apoptosis and release antimicrobial peptides such as phospholipases, Cathelicidin LL-37 that perform parasiticidal activity. Dendritic cells of the intestine induce adaptive immunity in the early steps and act as active effectors and immune sentinels. *C.parvum* infection is known to induce a Th1 immune response with IFN-*γ* production by CD4^+^ T cells [85]. Cryptosporidiosis will elicit an antibody response of IgM, IgG and Ig A [86]. The functions of innate and adaptive immunity are mentioned in Table 1. Because of the active immune response in immunocompetent individuals, *Cryptosporidium* infection would be mild to moderate or often asymptomatic and self-limiting [83]. But, due to faulty function in immunity in immunocompromised patients, the infection can become life-threatening and chronic [99].

Correlations between lack of immunity and *Cryptosporidium* infection have been reported in several studies; Cryptosporidiosis and Mannose-Binding Lectin (MBL) deficiency in AIDS patients, *mbl2* gene structural mutation in AIDS patients [95], C-X-C motif chemokine ligand 8 defect in malnutrition children [103–105], and low 4 (CD4^+^) T-cell counts in HIV/AIDS patients [52]. A study reported that the clinical condition of HIV patients with diarrhea who had CD4^+^ T-lymphocyte cell count less than 50 cell/mm^3^ was worse than the patients who had CD4^+^ T-lymphocyte cell count less than 100 cell/mm^3^ [106]. A study done in India revealed that IgG and IgA response for cryptosporidiosis occurred in both HIV positive and negative patients, suggesting that the HIV status may not play a role in modulation of specific antibody response [86]. Stimulating innate and adaptive immunity accordingly can be a promising therapy in controlling *Cryptosporidium* infection.

### 3.8. Diagnosis

#### 3.8.1. Sample Collection

*Cryptosporidium* species can be detected from unpreserved and preserved stool samples. The samples which will be delayed should be preserved in 10% formalin, polyvinyl alcohol, or sodium acetate formalin [107]. Before carrying out the microscopic examination, concentrating the stool sample is important to increase the sensitivity of microscopic examination by maximum oocysts recovery [107, 108]. Commonly used methods to concentrate are centrifugation, saturated salt floatation, Allen and Ridley's formol-ether method, and Sheather's sucrose flotation method [109, 110]. Formalin-ethyl acetate or formol-ether sedimentation method is a highly recommended stool concentrating method in clinical laboratory settings because it is easier to perform and preserve specimens [37, 44, 111].

#### 3.8.2. Microscopy

The wet mount has been used to identify *Cryptosporidium* oocysts of 4–6 *μ*m in diameter for initial screening in many studies [6]. Acid-fast modified Ziehl–Neelsen (mZN) staining technique has been used widely in many studies to observe oocysts stained in red on an unconcentrated fecal smear [17, 37, 39, 54, 68, 112]. As mZN staining only stains the internal structures (sporozoites) of the oocysts and leaves the remaining unstained. Empty oocysts without sporozoites present are left unstained and undetected, thus, reduces the sensitivity (41.2%) [107]. Auramine-phenol stain or safranin-methylene blue can be used as an alternative for mZN stain [18, 113]. Auramine-phenol staining is more sensitive and specific and is considered a gold standard method. This is used as a simple screening method [18]. Modified Kinyoun and Modified Trichrome staining, and Giemsa stain are also used to identify *Cryptosporidium* species [40]. However, these methods cannot distinguish between *Cryptosporidium* species [114].

#### 3.8.3. Immunological Methods

Immunological methods are based on antibody detection or antigen detection. The sensitivity and the specificity of these methods were found to range from 93% –100%. Acute infections are diagnosed by antigen detection, and sero-epidemiological surveys are carried out by antibody detection [107]. The antigen detection method consists of antibodies labeled with fluorescent reporters or enzyme reporters [107]. Monoclonal antibodies (mAbs) labeled with fluorescent reporters, which can be used to identify the epitopes on *Cryptosporidium* oocysts surface, and commercially produced mAbs are mostly used against *C. parvum* [115]. Antibodies labeled with enzyme reporters are available for enzyme immunoassay (EIA), immunochromatographic (IC) formats, and enzyme-linked immunosorbent assay (ELISA). These tests give specificity of 75%–100% and sensitivity of 92%–95.3% [18, 61, 116–118].

#### 3.8.4. Molecular Methods

Polymerase Chain Reaction (PCR) based methods are more sensitive and specific (100%) compared to microscopy and immunological assays to detect *Cryptosporidium* infection [6, 17, 18, 116]. Currently used PCR-based methods are nested-PCR, DNA hybridization, Restriction Fragment length Polymorphism PCR (PCR-RFLP), real-time PCR (rt-PCR), and multiplex-PCR [13, 15, 17, 19, 38, 118]. PCR-based methods use genetic markers to amplify the specific fragments of the *Cryptosporidium* genome. Commonly targeted sequences in the studies are small subunit (SSU) rRNA (*18S rRNA* gene), 60-kDa glycoprotein (*gp60*) gene, 18S rDNA, 70-kDa heat shock protein (*hsp70*) gene, internal transcribed spacer 1 (ITS1) region of rRNA, oocysts wall protein (*cowp*) gene, and actin gene [119–121]. In PCR-RFLP commonly used endonucleases to cleave PCR products are *SspI*, *VspI*, and *DdeI*. *SspI* and *VspI* differentiate *C. parvum*, *C. hominis, Cryptosporidium canis, Cryptosporidium serpentis,* and *C. meleagridis* [15, 122]. The real-time PCR technique diagnoses *Cryptosporidium* species by the use of the melting curve alone. The amplified product with the size of 272 bp enables this protocol to be more specific and sensitive [17]. Using TaqMan probes for *18S rRNA* gene in real-time PCR gives a higher efficiency [123–125]. Direct sequencing is carried out to evaluate the speciation, and 18S rRNA is commonly used for this purpose. Sequencing is often carried out by ABI BigDye Terminator v. 3.1 Cycle sequencing Kit [15, 119, 120].

### 3.9. Treatment

Spontaneous recovery is observed in immunocompetent individuals from *Cryptosporidium* infection, despite prolonged illness. But supportive therapy is needed [126]. Nitazoxanide is the drug approved by Food and Drug Administration to treat Cryptosporidiosis in immunocompetent patients. But it does not reduce the symptoms successfully all the time and shows only marginal results in malnourished children and HIV positives patients [127, 128]. Paromomycin, Rifaximin, or Azithromycin also can be used against *Cryptosporidium* infection [126]. In immunocompromised patients, *Cryptosporidium* infection cannot be eliminated by the host naturally due to the failure in immune reconstitution [127, 129]. Drugs that target metabolic pathways of *Cryptosporidium* and Ca^2+^ dependent protein kinases and microtubule function can suppress the growth of *Cryptosporidium* species [127]. Activating T lymphocytes, INF-*γ*, macrophages, CD40 ligand route can result in improving cellular immunity to resolve the symptoms in immunocompromised patients [4].

For patients with HIV infection, highly active antiretroviral therapy (HAART) can provide a complete cure for clinical symptoms by increasing the CD4^+^ lymphocytes to 200–300 cells/mm^3^ [129, 130]. It increases the expression of INF-*γ*, IL-15, and IL-4 [59]. HAART has a protease inhibitor that interferes directly with the *C. parvum* life cycle to reduce the shedding of oocytes and the intracellular parasites [130]. But some patients may not show improvement with HAART. For these patients, supportive therapy that includes rehydration therapy, antimotility agents, and electrolyte replacement can be an alternative until the development of better treatment [129]. Few studies showed that combination therapy including Paromomycin and Azithromycin facilitates a complete recovery in renal transplant recipients [13, 130]. Another study reports that the renal transplant recipients responded well to Paromomycin and antimotility agents [131]. However, the low number of studies has not permitted a solid basis for conclusion. If proper treatment was available for post-transplant and cancer patients infected with *Cryptosporidium* infection, immunosuppression therapy would not have to be disrupted. Among immunocompromised individuals, prevention is the most effective protective strategy [41].

### 3.10. Prevention

Appropriate measures for prevention are crucial among the patient population [3]. But there is not much evidence for prevention of *Cryptosporidium* infection [13, 40, 129]. The available guidelines are mostly based on observational studies and the opinion of experts. These include washing hands with soap, avoiding accidental intake of swimming pool, lake, and river water, avoiding contact with animal or human stool, and ensuring a safe drinking water by removal of oocytes, use of prophylactic agents such as bovine colostrum [3, 21, 129, 132]. Furthermore, identifying the areas under threat with potential reservoir populations will help in prevention [133].

## 4. Conclusion

*Cryptosporidium* infection is highly prevalent in immunocompromised, thus can be chronic and in the worst-case, lead to death. Common clinical presentations observed are chronic diarrhea, transient diarrhea, relapsing illness, weight loss, abdominal pain, vomiting, fatigue, and cholera-like disease. A high prevalence of cryptosporidiosis in immunocompromised patients is recorded in Asia, Africa, Europe, and North America. The immunity of the host is the main factor that decides the probability and the severity of infection. Most of the studies have observed decreased CD4^+^ T-cell count, poor hygiene, unsafe drinking water, high rainfall and diarrhoea among family members as risk factors for *Cryptosporidium* infection among immunocompromised PCR is a frequently used method along with immunological and/or staining procedures. Practicing qPCR and rt-PCR will give a precise and accurate diagnosis. As cryptosporidiosis cannot be eliminated naturally by the host, drugs that can suppress the growth of *Cryptosporidium* species and activate host immunity must be administered. These drugs increase the expression of INF-*γ*, IL-15, and IL-4 and activate T lymphocytes, INF-*γ*, macrophages, CD40 ligand route. Prevention of Cryptosporidiosis in immunocompromised is of vital importance.

## Figures and Tables

**Table 1:** Function of innate and adaptive immunity in patients with cryptosporidiosis.

	Structures/molecules	Functions	References
Innate immunity	Intestinal epithelial cells	Function as a physical barrier	[87]
		Expression of microbe recognizing pattern-recognition receptors (TLRs and NLR)	[88]
		Introduction of chemokines and proinflammatory cytokines expression	[88]
		Production of dendritic cells which attract chemokines	[89]
		Cellular source of producing IL-8	[87]
	IFN-*γ* (secreted by non-T-cell)	Stimulation of macrophages and produced iNOS-generated NO to activate the stress signaling cascade that includes the JNK pathway	[90]
	Phagocytes and NO	Protection from invading microorganisms	[91]
	Dendritic cells	Attract chemokines, express IFN-*α*/*β*	[89]
	Complement system (part of humoral innate immune system)	Production of complement factors MBL	[89]
	TLR-mediated pathways	Recognition of PAMPs, recruiting cascade of kinases by MyD88 NF-*κ*B nuclear translocation	[92]
		Expression of proinflammatory cytokine genes and initiation of host immune responses	[93]
	Antimicrobial peptides	Intestinal mucosal barrier component (cathelicidins and *α* and *β* defensins) Recruition of dendritic cells and T-cells	[94]
	MBL	Bind to carbohydrate residues which are on *Cryptosporidium*	[95]
		Activation of the lectin complement pathway. Opsonization and phagocytosis promotion	
		Lead the membrane attack complex formation	
	Chemokines	Interaction with G-protein-linked transmembrane chemokine receptors. Induction of chemotaxis and activation of leukocytes	[96]
	Cytokines	Modulation of innate and adaptive immune responses	[97]
	Prostaglandins	Upregulation of epithelial cell mucin production	[98]

Adaptive immunity	T-lymphocytes	Recovery from *Cryptosporidium* infection	[99]
	Helper T-cells	Th1 cells-secretion of IFN-*γ* and TFN-*α*	[100]
		Th2 cells-secretion of IL-4, IL-5, IL-10, and IL-13 and activation of B-cells to produce antibodies. CD4^+^ helper cells resolve the established *C. parvum* infection	
	IFN-*γ* (secreted by T-cells)	Prevention of initiation of infection and limits the infection. Mediation of CD4^+^ response	[100]
	TNF-*α*	Stimulation of prostaglandin production	[101]
	B-cells	Production of parasite specific immunoglobulin	[102]

Abbreviations: CD4, cluster of differentiation 4; iNOS, inducible nitric oxide synthase; IFN-*α*/*β*, interferon alpha/beta; IFN-*γ*, interferon gamma; IL-4, interleukin 8; IL-5, interleukin 5; IL-8, interleukin 8; IL-10, interleukin 10; IL-13, interleukin 13; JNK, c-Jun N-terminal kinase; MBL, mannose binding lectin; NO, nitric oxide; NLR, Nod-like receptors; NF-*κ*B, nuclear factor kappa light chain enhancer of activated B cells; PAMPs, pathogen-associated molecular patterns; Th1, T helper cell type 1; Th2, T helper cell type 2; TLRs, Toll-like receptors; TFN-*α*, tumor necrosis factor alpha.

## Data Availability

All data supporting this comprehensive review are from previously published studies and have been cited.

## Nomenclature

CD4: Cluster of differentiation 4
cowp: Oocysts wall protein
DNA: Deoxyribonucleic acid
DOCK 8: Dedicator of cytokinesis 8
EIA: Enzyme immunoassay
ELISA: Enzyme-linked immunosorbent assay
gp60: 60-kDa glycoprotein
HAART: Highly active antiretroviral therapy
HIV/AIDS: Human immunodeficiency virus/acquired immunodeficiency syndrome
HSCT: Hematopoietic stem cell transplant
IC: Immunochromatographic
IFN-*α*/*β*: Interferon alpha/beta
IFN-*γ*: Interferon gamma
IL-4: Interleukin 8
IL-5: Interleukin 5
IL-8: Interleukin 8
IL-10: Interleukin 10
IL-13: Interleukin 13
iNOS: Inducible nitric oxide synthase
ITS1: Internal transcribed spacer 1
JNK: c-jun-N-terminal kinase
kDa: Kilodalton
(hsp70): 70-kDa heat shock protein
mAbs: Monoclonal antibodies
MBL: Mannose Binding Lectin
mZN: Modified Ziehl–Neelsen
NF-*κ*B: Nuclear factor kappa light chain enhancer of activated B cells
NLR: Nod-like receptors
NO: Nitric oxide
PAMPs: Pathogen-associated molecular patterns
PCR: Polymerase chain reaction
PCR-RFLP: Restriction fragment length polymorphism PCR
rRNA: Ribosomal ribonucleic acid
rt-PCR: Real-time PCR
SUU: Small subunit
TFN-*α*: Tumor necrosis factor alpha
Th1: T helper cell type 1
Th2: T helper cell type 2
TLRs: Toll-like receptors
XHIM: X-linked hyper-IgM

## Supporting Information

Supporting Table: Included studies carried out on *Cryptosporidium* infection in immunocompromised patients in this review.
