# Supplementary material for: Cryptosporidiosis in a Zoonotic Gastrointestinal Disorder Perspective: Present Status, Risk Factors, Pathophysiology, and Treatment, Particularly in Immunocompromised Patients
Source: J Trop Med. 2024 Nov 5;2024:6439375. doi: 10.1155/2024/6439375 (PMC11557182; doi:10.1155/2024/6439375)
Supplement: Supporting Information — Additional supporting information can be found online in the Supporting Information section. [file 6439375.f1.doc]

**Supplementary Table . Included studies carried out on *Cryptosporidium* infection in immunocompromised patients in this review.**

| **Country** | **Year** | **Number and type of**  **Subjects** | **Test used in Diagnosis** | ***Cryptosporidium* infection rate** | **Risk factors** | **Reference** |
| --- | --- | --- | --- | --- | --- | --- |
| **HIV** | | | | | | |
| France | 2015-2017 | 210 immunosuppressed patients | rt-PCR  Gp60 sequencing | *C. parvum* – 66%, *C. hominis* – 22%  Organ transplant – 49%, HIV infection – 30%, Mortality rate – 6% | High rainfall period | [23] |
| Iran | 2014-2015 | 250  HIV patients | Nested PCR followed by RFLP (amplified 18s rRNA) | 10.8% (27/250) positive for different *Cryptosporidium* spp.  *C. parvum* – 70.38%, *C. hominis* – 25.92% | CD4+ T-cell count < 200 cells/μl ((p=0.000),  HIV infection | [19] |
| Nigeria | - | 52  HIV infected children | ELISA and western blot in children over 18 months and DNA PCR for children aged less than 18 months | Overall infection – 10/52 (19.2%) |  | [16] |
| Thailand | 1999-2004 | 155  HIV infected patients | 18s rDNA locus sequencing | *C. parvum –* 5/155  *C. hominis –* 42/155 |  | [22] |
| Iran | 2013-2014 | 350  Immunocompromised patients |  | Overall infection 3/350  HIV/AIDS patients – 2  Organ transplant patients – 1 |  | [21] |
| India |  | Total 409,  206 HIV seropositive, 153 seronegative patients with diarrhea and, 50 healthy individuals | ELISA  PCR | Healthy control was not found with *Cryptosporidium* infection  ELISA - Seropositive 39/206 (18.9%), seronegative 21/153 (13.7%)  PCR – 27/206 (13.1%), 17/153 (11.1%) | CD4 count < 200 | [18] |
| Ethiopia | 2009-2011 | 520  HIV/AIDS | PRC-RFLP analysis of SSU rRNA gene | *C. parvum* – 92 patients  *C. hominis* – 25 patients | Calf contact | [15] |
| North India |  | 154 HIV seropositive subjects  50 HIV negative controls | Microscopy, fecal cultures, and serological tests,  FACS count | *C. parvum* – 60.42% |  | [121] |
| Republic of Congo | 2009-2012 | 242  HIV patients | ZN staining, rt-PCR, nested PCR-RFLP and PCR amplification and sequencing | *Cryptosporidium* spp. – 5.4%  *C. parvum* – 0.4%  *C. hominis* – 1.6%  Diarrhea, low CD4+ counts of < 100 cells/mm^3^ |  | [17] |
| Iran | 2008-2009 | 183 immunocompromised patients  HIV infection, hematological malignancy, used of immunosuppressive drugs after solid transplant | mZN staining, 18S rRNA gene amplification and sequencing | Overall *Cryptosporidium* infection – 6%.  *C. parvum* - HIV infected – 5/183, bone marrow transplant – 1/183, chronic lymphocytic leukemia – 1/183.  *C. hominis* – HIV infected – 2/183, acute lymphocytic leukemia – 2/183, | Univariate analysis –risk factors are diarrhea, CD4+ lymphocytes less than 100 cells/mm^3^, other microbial infection, weight loss, abdominal pain, highly active antiretroviral therapy, and diarrhea in household members.  In multivariate analysis – risk factors are CD4+ lymphocytes less than 100 cells/mm^3^, and diarrhea in household members | [20] |
| Ghana | 2018 -2019 | 418 HIV patients | Cryptosporidium antigen detection by ELISA | *C. parvum* - 6.2% | use of public water closet toilet facilities | [122] |
| Honduras | 2019- 2020 | 102 patients living with HIV | microscopy, acid-fast stained smears, and a rapid lateral flow immunochromatographic test. | *C. parvum* - 4/102 (3.92%) |  | [123] |
| Algeria | 2016 to 2018 | 350 HIV/AIDS patients | mZN staining  PCR | *C. parvum-33/350* |  | [124] |
| Nigeria |  | 196 HIV positive patients | mZN | *C. parvum-* 10/196 | CD4 >350 | [59] |
| Egypt | 2019 May-2021 May | 150 HIV positive patients | mZN | *C. parvum- 66*/150 | rural residence, illiteracy, diarrhea and CD4+ T-cell counts <200 cells/μl | [125] |
| Ethiopia | 2018 | 304 HIV/AIDS cases before and after commencement of antiretroviral therapy. | mZN | *Before ART- Cryptosporidium* spp-58/304  *After ART- Cryptosporidium* spp- 24/304 | lower <500 cells/mm^3^ CD4^+^ cell counts  Drinking unsafe water, poor knowledge on HIV | [126] |
| Iran | 2020 | 26 HIV patients,  11 Organ transplant patients | mZN | *Cryptosporidium* spp- 7-26 (26.92%)  *Cryptosporidium* spp- 2/11 (18.18%) |  | [127] |
| **Organ transplant** | | | | | | |
| Iran | 2009-2010 | 176  Immunocompromised patients | Coproantigen test using ELISA | Children suffered from HM– 4.2%, Adult cancer patients – 4.0%, RT recipients – 4.5% | Frequent among HIV^+^ patients | [28] |
| France | 2015-2017 | 210 | rt-PCR  Gp60 sequencing | *C. parvum* – 66%, *C. hominis* – 22%  Organ transplant – 49%, HIV infection – 30%, Mortality rate – 6% | High rainfall period | [23] |
| Turkey | 2012-2015 | 62 immunocompromised children undergoing transplant or malignancy | Microscopy and staining | 22.5% (14/62) of the subjects showed infection |  | [32] |
| Iran | 2016-2017 | 85 cancer patients  25 organ transplants  80 primary immunodeficiency | ZN staining  Nested PCR | Primary immunodeficiency – 6/80  Organ transplant – 6/25 |  | [24] |
| India |  | 358  200 (control)  Renal transplant recipient | PCR-RFLP | RT recipient – 30/358, Healthy controls – 0/200, With diarrhea – 26/162, Without diarrhea – 4/162, *C.parvum –* 10/30, *C.hominis* – 20/30 |  | [27] |
| France | 2006-2010 | 47 solid organ transplants  KT – 41, Liver/Kidney – 1, Liver – 1, Heart – 3, Pancrease - 1 | ZN staining | All 47 patients had cryptosporidiosis.  CD40 ligand deficiency – 1, HIV - 4 | Environmental risk factors like rural areas, contact with farm animals, drinking non-potable water, recreational water usage, traveling to endemic areas | [26] |
| Hungary | 2015 | 191 Pediatric organ transplant patients (49) and their family | Kinyoun staining | 18% (35/191) infection |  | [29] |
| Iran | 2013-2014 | 350 Immunocompromised patients | ZN staining | Overall infection 3/350, HIV/AIDS patients – 2  Organ transplant patients – 1 |  | [21] |
| USA | 2012-2013 | 422  Organ transplant | PCR, Plasma CMV DNA qPCR, Stool PCR, stool, enzyme immunoassay | Overall infection – 160/422, KT– 42.7%, Liver transplant – 24.6%, Heart transplant – 11.1%, Pancreases transplant – 2.6% |  | [25] |
| Sudan | 2012-2013 | 300 RT recipient  300 (control) | mZN staining | Overall infection – 1.2% (7/600)  Transplant recipient – 5, Control – 2 |  | [30] |
| Yemen | 2016- 2017 | 35 cancer patients  3 Renal transplant patients | mZN | *Cryptosporidium* spp- 26/35  *Cryptosporidium* spp-3/3 | Rural area  drinking from piped water  Contact with animals  Poor hygiene | [128] |
| **Cancer** | | | | | | |
| Iran | 2009-2010 | 176 Immunocompromised patients | Coproantigen test using ELISA | Children having HM– 4.2%, Adult cancer patients – 4.0%, RT recipients – 4.5% | Frequent among HIV^+^ patients | [28] |
| Turkey | 2012-2015 | 62 immunocompromised children undergoing transplant or malignancy | Microscopy and staining | 22.5% (14/62) of the subjects showed infection |  | [32] |
| China | 2018 | 195 patients with digestive malignancies before chemotherapy and in control population | Nested PCR (amplified 18s rRNA) | Colorectal cancer – 17.24% (20/116), esophageal cancer – 6.25% (1/16), liver cancer – 14.29% (1/7), small intestine cancer – 40% (2/5), gastric cancer – 4% (2/51), overall infection – 26/195 | Except for gastric cancer, all gastro intestinal cancers were significantly associated with *Cryptosporidium* spp. Infection | [13] |
| Poland | 2009-2010 | 87 colorectal cancer patients | Immunoenzymatic tests | Overall infection – 11/87 (12.6%) | No statistical correlation with gender, age, neoplasm advancement stage, differentiation star or tumor localization | [34] |
| Lebanese | 2012-2013 | Total of 218 individuals | PCR, microscopic observation, IFA | Overall infection – 15/72 (21%) | Colon adenocarcinoma (p=0.003) | [33] |
| Egypt | 2013-2013 | 150 with chronic liver diseases and diarrhea, 50 with diarrhea | mZN staining, ELISA | *Cryptosporidium* infection in chronic liver disease – 45/150 (30%) |  | [129] |
| Poland | 2009-2014 | 108 consecutive patients with colorectal cancer, 125 (control group) | Immunoenzymatic test | Overall infection - 14/108 (13%) | Male gender | [130] |
| Iran | 2014-2015 | 330 hemodialysis patients  150 healthy individuals | mZN staining | *Cryptosporidium* infection – 10/330 (3%), 1/150 (1%) | Place of residence, hygiene status, education level, diarrhea, abdominal pain (p<0.05). Age (p=0.003), under 20 years. | [31] |
| Iran | 2008-2009 | 183 immunocompromised patients (HIV infection, hematological malignancy, used of immunosuppressive drugs after solid transplant) | mZN staining, 18S rRNA gene amplification and sequencing | Overall *Cryptosporidium* infection – 6%.  *C. parvum* - HIV infected – 5/183, bone marrow transplant – 1/183, chronic lymphocytic leukemia – 1/183. *C. hominis* – HIV infected – 2/183, acute lymphocytic leukemia – 2/183, | In multivariate analysis – significant risk factors are CD4+ lymphocytes less than 100 cells/mm^3^ and diarrhea in household members | [21] |
| Poland | 2007-2016 | 148 hematological malignancy patients  101 healthy individuals | Modified Kinyoun and Modified Trichrome staining  PCR-RFLP | *Cryptosporidium* infection in HM patients – 3%, Healthy individuals – 0% (*p*=0.02) | Male, consuming unfiltered water | [27] |
| United Arab Emirates | 1999-2000 | 140 children with diarrhea | mZN staining | *Cryptosporidium* infection in – 4/140  Healthy children – 3/140 (Cured without medication) |  | [61] |
| Iran | 2015-2016 | 132 Children with Cancer  132 non-cancer control | mZN staining  PCR | *Cryptosporidium* infection in children with cancer |  | [8] |
| Poland | 2009-2010 | 145 non-HIV infected colorectal cancer patients | Immunofluorescence antibody test  Genus-specific nested PCR  SSU rRNA sequencing | *Cryptosporidium meleagridis* infection – 1/145 | Smoking and alcohol consumption | [62] |
| Yeman | 2020 | 30 patiens with cancer | mZN staining | *C. parvum-*27/30 (90%) | Residing in a rural area, Consumption of water from wells | [128] |
| Iran | 2014-2020 | 187 Cancer patients | mZN | *Cryptosporidium* spp-8/187 |  | [131] |
| Iran | 2019- 2020 | 87 colorectal cancer patients | mZN, nested PCR | *Cryptosporidium* spp-37/87 | residence in urban areas | [132] |
| Egypt | 2018 | 145 children with cancer | mZN, nested PCR | *Cryptosporidium* spp-43/145 |  | [133] |
| **Primary Immunodeficiency** | | | | | | |
| Iran | 2016-2017 | 85 cancer patients, 25 organ transplants, 80 primary immunodeficiency | ZN Staining  Nested PCR | Primary immunodeficiency – 6/80  Organ transplant – 6/25 |  | [24] |
| Netherlands | 1980-2006 | 6 XHIM syndrome type 1 and primary CD4 lymphopenia patients | mZN Staining, IFA, Molecular analysis with genotyping | *Cryptosporidium* species in 3 XHIM patients and 1 primary CD4 lymphopenia |  | [4] |
| Egypt | 2016- 2017 | 50 children with Primary immunodeficiency  52 children with secondary immunodeficiency | mZN, nested PCR  mZN, nested PCR | *Cryptosporidium* spp- 8/50  *Cryptosporidium* spp 14/52 | Summer season | [134] |
| HIV: human immunodeficiency virus rt-PCR: real-time PCR; gp60: 60-kDa glycoprotein; RFLP: restriction fragment length polymorphism; CD4: cluster of differentiation 4; ELISA: enzyme-linked immunosorbent assay; DNA: deoxyribonucleic acid; rRNA: ribosomal ribonucleic acid; SUU: small subunit; rDNA: ribosomal deoxyribonucleic acid; FACS: fluorescence-activated single cell sorting; mZN: modified Ziehl-Neelsen; CMV DNA qPCR: Cytomegalovirus DNA quantitative PCR; EIA: enzyme immunoassay; IFA: immunofluorescence assay; AIDS: acquired immunodeficiency syndrome: RT: renal transplant; HM: hematopoietic malignancy; XHIM: X-linked hyper-IgM | | | | | | |
